# Prevalence, Intensity, and Factors Associated with Urogenital Schistosomiasis among Women of Reproductive Age in Mbogwe District Council, Geita Region, Tanzania

**DOI:** 10.1155/2020/5923025

**Published:** 2020-10-23

**Authors:** Erasto Eleck Rite, Secilia Ng'weshemi Kapalata, David Zadock Munisi

**Affiliations:** ^1^Department of Public Health, College of Health Sciences, University of Dodoma, P.O. Box 259, Dodoma, Tanzania; ^2^Department of Biomedical Sciences, College of Health Sciences, University of Dodoma, P.O. Box 259, Dodoma, Tanzania

## Abstract

**Background:**

Urogenital schistosomiasis remains a public health problem in Tanzania. Control programs mostly target school-going children ignoring other vulnerable groups like women of child bearing age. Previous evidence has shown that women of reproductive age suffer greatest morbidities in endemic areas. This study sought to determine the prevalence, intensity, and factors associated with urogenital schistosomiasis among women of reproductive age in the Mbogwe District.

**Methods:**

A population-based analytical cross-section study was conducted in the Mbogwe District. A semistructured questionnaire was administered. Urine samples of 20-30 mils collected between 10.00 am to 02.00 pm and examined for Schistosoma eggs and infection intensity microscopically. Data analysis was done using SPSS version 20.

**Results:**

A total of 426 women of reproductive age, with median age of 26, and interquartile range of 11years were recruited and assessed. The prevalence of urogenital schistosomiasis was 4.5% and mean egg intensity of 19.5eggs/10mil of urine. After adjusting for confounders, lower level of education was associated with an increased risk of urogenital schistosomiasis infections (AOR 8.355, 95% CI 3.055-23.001).

**Conclusion:**

Urogenital schistosomiasis among women of reproductive age in the Mbogwe District is a problem. Education is the factor associated with the disease; the neglected tropical disease control program should develop strategies that should include provision of health education and should involve women of reproductive age as they act as infection reservoir. More studies are recommended to explore the possibility of reproductive complications among infected women in endemic areas.

## 1. Background

Schistosomiasis also known as bilharzia or snail fever is an infectious disease caused by trematode parasitic worms of the genus Schistosoma. There are three main species under the genus Schistosoma that can infect humans, and these are *Schistosoma haematobium*, *Schistosoma mansoni*, and *Schistosoma japonicum* [1]. Three other species are locally distributed and less common which are *Schistosoma mekongi,* which is found in the Mekong River basin as well as *Schistosoma guineensis* and *Schistosoma intercalatum,* both found in west and central Africa [2]. The disease is the second leading endemic parasitic disease after malaria on the list of parasitic diseases in 76 countries worldwide that affects an estimated 250 million people and about 779 million people are at risk of infection, 93% of which occurs in sub-Saharan Africa, whereby the United Republic of Tanzania rank second to Nigeria in terms of disease burden with prevalence estimates ranging between 12.7% and 87.6% with major agents being *Schistosoma haematobium and Schistosoma mansoni* [3–5]. The disease is further implicated to be the cause of over 280,000 death each year in Africa alone [6].

Schistosomiasis caused by *Schistosoma haematobium* affect nearly 45 million women living in sub-Saharan Africa. The adults of these worms dwell in blood vessels surrounding the bladder and genital tract of female, and the female worms lay eggs intravascularly, resulting into chronic granulomatous inflammation in urinary bladder, ureter, cervix, and vaginal wall. Because the urinary and genital tracts are almost always both affected, the WHO has renamed this disease urogenital schistosomiasis, with presence of *Schistosoma haematobium* eggs in urine or genital tract being diagnostic [7]. On women, urogenital schistosomiasis has an effect on reproductive health characterizing a condition known as female genital schistosomiasis which is attributed to occurrence of Schistosome eggs and worms in the genital organs, with signs and symptoms like vaginal itching, vaginal discharge, postcoital bleeding, genital pelvic discomfort, infertility, and lesion of the cervix and vaginal [7]. Passage of Schistosoma ova in urine has been observed to be associated with features of sandy patches, lesions in cervix, and vaginal wall among women from *Schistosoma haematobium* endemic areas [8]. *Schistosoma haematobium* adults inhabit blood vessels that surrounds the urinary bladder and female genital tract and lay eggs that migrate through tissue of nearby organs, causing chronic inflammation most commonly in the urinary bladder, ureters, cervix, uterus, fallopian tube, ovaries, and vagina resulting into uterine enlargement, menstrual disorder, cervicitis, infertility, and high rate of developing spontaneous abortion and the risk of ectopic pregnancy [9, 10]. However, despite all these reproductive complications, women of reproductive age remain to be an understudied subpopulation in endemic areas particularly in sub-Saharan Africa [11]. Furthermore, the control of Schistosomiasis in endemic countries is based on preventive chemotherapy using Plaziquantel 40 mg/kg applied to school-aged children excluding pregnant women who are also likely to suffer severe morbidities that affect their overall reproductive health [12].

In order for women of reproductive age to be included in the national schistosomiasis control programs in endemic countries, there is a need of having enough epidemiological evidence quantifying the burden of infection and their associated factors. This study was therefore designed to determine the prevalence, intensity, and factors associated with urogenital schistosomiasis among women of reproductive age in the Mbogwe District Council, Geita Region, Tanzania.

## 2. Methods

### 2.1. Study Area

The study was conducted in the Mbogwe District council in the Geita Region. Mbogwe District is in the Western apex of the Shinyanga Region, and it lies between longitudes 31-32° East and latitudes 3-3.30° South [13]. The district boarders Geita and Chato districts of the Geita region in the North West. In the eastern boundary lies the Kahama district of the Shinyanga region while the southern boundary is covered by the Urambo district in the Tabora region and in west lies the Bukombe district of the Geita region [13]. Administratively, the district is divided into three divisions, namely, Masumbwe, Mbogwe, and Ilolangulu in which there are 17 wards, 86 villages, and 334 hamlets [13].

Generally, about 90% of the population depends on agriculture and livestock keeping as their major sources of income. The major food crops grown include maize, rice, cassava, sweat potatoes, and varieties of leguminous crops and banana, while cotton, tobacco, and sunflower are cash crops [13].

The district has a tropical type of climate with a mean annual temperature of 22°C and an average annual rainfall of between 900 and 1200 mm. The district has a bimodal rainfall character, and rainfall is fairly evenly distributed with short rains from September to December followed by dry spell from January to February before long and heavy rains set in between the months of March and May [13].

### 2.2. Study Design

This was a population-based analytical cross-sectional study with the quantitative approach conducted in the Mbogwe District from May to June 2019.

### 2.3. Study Population

The study involved women of reproductive age (15-49years) in the selected households who were permanent residents of the Mbogwe District council, and those who were available and agreed to participate in the study.

### 2.4. Sample Size Estimation

The sample size was calculated by assuming the estimate prevalence of 50% (as prevalence of urogenital schistosomiasis among women of reproductive age in endemic area range between 25 and 72.2% according to [14]). The estimate of the sample size was obtained using the formula below:

(1) $$\begin{array}{c} n=\frac{Z\alpha^{2}p\left(1-p\right)}{\delta^{2}}, \end{array}$$

where *Zα* = standard normal deviate at 95% confidence interval corresponding to 1.96; *P* = 50%. Assumed true population prevalence of urogenital schistosomiasis, *δ* = absolute error between the estimated and true population prevalence of urogenital schistosomiasis among women of reproductive age of 5% or 0.05 and *n* = estimated sample size.

(2) $$\begin{array}{c} n=\frac{1.96\text{x}1.96\text{x}0.5\left(1-0.5\right)}{0.5\text{x}0.5}. \end{array}$$

*n* = 384 is the study participants, adding up 10% nonresponse rate, and yields a minimum sample size of 427 study participants for this study.

### 2.5. Sampling Technique

Mbogwe district was selected purposively based on the geographical location, that is the Lake Zone, with high prevalence of schistosomiasis. Three wards Nhomolwa, Nanda, and Ushirika from two divisions masumbwe and mbogwe were randomly selected, and from each wards, half of the villages were randomly selected to make a total of 11 villages (Nhomolwa, Nyahwiga, and kabanga from Nhomolwa ward, Ushirika, Mlale, Ushetu, Buzigula and Kadoke from Ushirika ward, Nanda, Nyangholongo, Kisumo and Muungano from Nanda ward).

A total of 1014 households with 922 women of reproductive age were registered in Nhomolwa, Nyahwiga, and Kabanga village from the Nhomolwa ward and then, we randomly selected one participant in every 6^th^ household with women aged 15-49years until the required sample size was attained. At the Ushirika ward, five villages with 2070 households and 1860 women of reproductive age were selected. For inclusion in the study, we sampled every 13^th^ household with women aged 15-49years, then one women was randomly selected when there were more than one eligible women. The last ward was Nanda where four villages were randomly selected. The selected villages had a total of 1616 households with 1498 women of reproductive age. For inclusion into the study, we randomly selected one participant in every 10^th^ household with women of reproductive age until when the required sample size was attained.

### 2.6. Research Instruments

A pretested Kiswahili translated semistructured questionnaire was used to collect sociodemographic, economic information, and potential risk factors for *S. haematobiumi* infection in women of reproductive age. Microscopy was used to detect *Schistosoma haematobium* egg and infection intensity in urine samples collected from the study participants.

### 2.7. Ethical Approval and Consent to Participate

Ethical clearance (UDOM/DRP/134/VOL VII) was obtained from the University of Dodoma, Directorate of research and publication and administration authorization from district executive director of Mbogwe (MDC/ADM/S.20/17 VOLL.4/151). Written and verbal consent was obtaining before enrollment into the study. For participants who were below 18 years of age, parents or guardians provided consent, and assent was sought from the respondents themselves. Participation was voluntary, and the collected data were kept anonymous and confidential with access being limited only to the lead investigators.

### 2.8. Parasitological Process

Sterile containers with the participants' ID number were used to collect urine sample of 20-30 mils between 10 am and 2 pm fixed with 10% formalin and place them in the cold box and transfer to the district laboratory Masumbwe hospital. Parasitological analysis was performed by experienced laboratory technicians at the Masumbwi district hospital laboratory. Urine sample was syringed and filtered using filter membrane and place on glass slide for examination of *Schistosoma haematobium* eggs under the light microscope at ×40 magnification, sample found to have egg was recorded as positive for Schistosoma, and the number of eggs present in each specimen was counted and recorded for intensity. For quality assurance, a random sample of 10% of the negative and positive Kato-Katz thick smears was reexamined by a third technician. Those with positive findings of Schistosoma egg were treated with praziquantel 40 mg/kg free of charge.

### 2.9. Data Entry, Cleaning, and Analysis

Data entry, cleaning, and analysis was done using SPSS (version 20). Descriptive statistics, including frequencies, percentages, median, interquartile, range and mean values, were used to visualize the data. Intensity of infection was calculated using geometric mean intensity (GMI), and intensities were classified into two categories of light infection (<50eggs/10mil of urine) and heavy infection (>50eggs/10mil of urine) and for reducing the impact of zero egg count, and normalization of the data geometric mean of egg output was calculated in infected women only. The statistical test performed included bivariate analysis to identify the factors associated with *Schistosoma haematobium* infection to be included in the multivariate logistic regression for analysis of risk factors for variable that had *p* value less or equal to 0.2.

## 3. Results

### 3.1. Social Demographic and Economic Profile of Respondents

**Figure 1:**
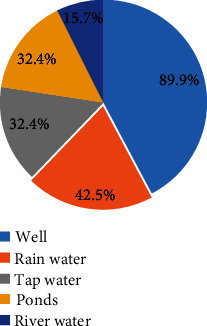
Distribution of water source for domestic use (*n* = 426).

A total of 426 participants were enrolled in this study. The age of the participants ranged from 15 to 49 years with a median of 26 years and interquartile range of 11 years. Majority of the respondents 323 (75.8%) were aged between 15 and 30 years. Most respondents 340 (79.8%) were married or cohabiting. The study further found that most of the participants 340 (79.8%) had attained primary level of education or above, and the remaining 86 (20.2%) had not attended any formal training. Majority 375 (88%) of the participants were unemployed and were involved in either farming activities or petty business, and others were just housewives and students. The main source for water for domestic use among study participants was tap water 383 (89.9%), followed by rain water 181 (42.5%) and while very few participants mentioned to use water from the river 67(15.7%) (Figure 1).

Fetching water was the main reason for participants to contact water source 387 (90.8%), followed by washing 352 (82.6%), agriculture activities 323 (75.8%), swimming 188 (44.1%), and the last is fishing 89 (20.9%). Majority of participants 422 (99.1%) had latrine at their home place (Table 1).

### 3.2. Prevalence of Urogenital Schistosomiasis

Of all the 426 participants who were screened for urogenital schistosomiasis in this study, 4.5% (95% CI: 0.025–0.064) were found to be infected, and the Ushirika ward had the highest 7.04% (95% CI: 0.661–0.747) prevalence of infection as compared to Nanda and Nhomolwa wards which had 4.22% (95% CI: 0.375–0.469) and 2.11% (95% CI: 0.172–0.25), respectively. The prevalence was further observed to vary across different age groups, and it was observed that respondents of the age between 15 and 30 years having the highest 5.30%(95% CI: 0.483–0.577) prevalence of infection, followed by 1.90% (95% CI: 0.153–0.227) in the 31-49 years' age group (Table 2).

### 3.3. Intensity of Urogenital Schistosomiasis among Women of Reproductive Age

The intensity of infection among study participants ranged from 8 to 38 eggs/10mil of urine with the overall geometric mean of 19.5 eggs/10mil of urine with 95% CI (14.9515-23.0484). According to the WHO categorization of *S.haematobium* infection intensities, all infected participants had light infection intensities, i.e., <50 eggs/10mil of urine. The findings also summarize that there was no significant difference between geometric mean egg intensity across ward, age groups, education level, occupation, source of water for domestic use, and activities that led to contacting water bodies.

### 3.4. Factors Associated with Urogenital Schistosomiasis among Study Participants

Binary logistic regression analysis was performed to determine the relationship between urogenital schistosomiasis and different socioeconomic and demographic characteristics among study respondents. The findings show that level of education and source of water for domestic use (river) were significantly associated with urogenital schistosomiasis (OR = 6.087, *p* < 0.001) (OR = 3.374, *p* = 0.014), respectively (Table 3).

Multivariate regression analysis was then performed after controlling for age, source of water for domestic use, and reason for contacting water body, and it was observed that level of education was significantly associated with urogenital schistosomiasis, whereby women with no formal education had 8.355 times higher odds of having urogenital schistosomiasis infection as compared to those who had primary and above education (*p* < 0.001, CI 3.035-23.001) (Table 4).

## 4. Discussion

In order for women of reproductive age to be included in the national schistosomiasis control programs in endemic countries, enough epidemiological evidence quantifying the burden of infection and their associated factors is necessary. Therefore, this study reports the prevalence, intensity, and factors associated with urogenital schistosomiasis among women of reproductive age in the Mbogwe District Council, Geita Region, Tanzania.

This study found that the overall prevalence of urogenital schistosomiasis infection among women of reproductive age in the study area was generally low (4.5%). This observed low prevalence of infection could be attributed to high coverage of latrine facility (99.1%) and high coverage of piped water supply in the studied communities. But the observed low prevalence might also be premised by the fact that there was high coverage of praziquantel mass treatment among school children in the area, much as children are the main reservoir of infection and they are the main source of environmental contamination with schistosoma eggs, and their treatment translates to reduction of transmission which has benefit even to untreated members of the community as seen in this study among women of child bearing age. This prevalence of infection was generally lower as compared to what was reported in the study conducted in the Mwanga district, Kilimanjaro region northern Tanzania where the prevalence was 36% [15] and in Volta basin of Ghana prevalence was 24.8% [14]. However, the prevalence reported in this study is more or less similar to what was reported in a study conducted in Sengerema and Misungwi district north-west of Tanzania where the prevalence was 5% (range from 0% to 11%) [5].

It is a general characteristic of helminths infections, schistosomiasis being one of them, that the prevalence of infection varies significantly from one place to another according to variation in exposure pattern even in places close to one another [5]. In this study, it was observed that Ushirika had relatively higher prevalence of infection as compared to other wards, with Nhomolwa bearing the least prevalence. The observed relatively higher prevalence of infection in the Ushirika ward is likely to be due to higher level of exposure to cercarial infested water bodies as a result of high engagement in activities such as farming and fishing in the ponds.

It is well known that the risk for schistosomiasis related morbidity is basically related to infection intensity [16, 17]. In this study, it was generally found that the overall mean egg intensity was light according to the WHO categorization of *S.haematobium* infection intensities, i.e., <50 eggs/10mil of urine [18]. However, these light infections need not be taken lightly as there is sufficient evidence to suggest that some forms of infection-associated morbidity are associated with the mere presence of infection. In other words, even light-intensity Schistosoma infection can be associated with significant patient morbidity [19]. The mean egg intensity found in this study is relatively higher as compared to that found in a community-based study in Tanzania where median intensity was 2.2 eggs/10mil of urine [20]. The slightly higher intensity found by this study could be because of the fact that the two studies studied different populations, with the community-based study involving school going children to whom most of the schistosomiasis control efforts are directed including annual preventive chemotherapy. However, the mean egg intensity reported in this study is relatively lower as compared to that reported in a study conducted in the western part of Madagascar where the median egg intensity was 76 egg/10 ml of urine [12]. This difference could be because of the difference on exposure pattern among women of reproductive age in these two study sites.

The study found that urogenital schistosomiasis was more common among women who had no formal education as compared to those with primary level of education and above. This observation is in line with other studies done elsewhere [21]. It has been suggested that, education may affect attitudes and behavior in different ways in different settings, with individuals with low educational status being more likely to cross a stream or river barefooted than their more educated counterparts [22]. Conversely, the self-awareness of the disease may account for the relatively lower risk of the disease among women with primary level of education and above [23]. This highlights the need for health education in endemic communities to lower the overall risk of acquiring the disease by raising community's level of awareness about the disease.

Among children, age has been known to be an important determinant of schistosomiasis [24]. However, in our study, we found that age had no influence on *Schistosoma haematobium* infection among women of reproductive age. This difference could be because of the difference on the study population in which case, age acts as an important predictor of Schistosoma infections among children, and it ceases in adulthood indicating similar exposure to infection across age groups in adulthood. Similar findings have also been reported by another study conducted in Nigeria [25]. Different results have also been reported elsewhere; in which case, women below 20 years were observed to have more infections that older women [26].

One major limitation of this study is the fact that we recruited the study participants proportionally in each village as while the population density was not the same in each recruitment site. We acknowledge that this could have resulted into unequal representation of the different study site. Another limitation is the fact that we took only one urine sample, and this might have underestimated the prevalence of urogenital schistosomiasis in our study.

## 5. Conclusion

The findings from this study indicate that generally, the prevalence and intensity of urogenital schistosomiasis is low, and lack of education was found to be a risk factor for the disease. High coverage of latrine ownership in the study area could partly explain the observed low prevalence and intensities of Schistosoma haematobium infection, but disease still remains a public health problem as those at risk groups are not included in national mass preventive chemotherapy. Further studies that will investigate the effect of *Schistosoma haematobium* infection of the reproductive health of women of reproductive age are highly recommended.

## Figures and Tables

**Table 1:** Social demographic and economic profile of respondents (*N* = 426).

Variable	*n*	Percentage
Age group (years)		
15-30	323	75.8
31-49	103	24.2
Marital status		
Married/cohabit	340	79.8
Not married	60	14.1
Divorced/widow	26	6.1
Education		
No formal education	86	20.2
Primary and above	340	79.8
Occupation		
Unemployed	375	88.0
Employed	51	12.0
Reason for contact water		
Agriculture	323	75.8
Fetching water	387	90.8
Fishing	89	20.9
Washing	352	82.6
Swimming	188	44.1
Latrine ownership		
Yes	422	99.1
No	4	0.9

**Table 2:** Prevalence of urogenital schistosomiasis in the study area (*N* = 426).

Variable	Tested (*n*)	Infected (*n*)	Prevalence %(95% CI)
Ward			
Nhomolwa	142	3	2.11% (95% CI: 0.172–0.25)
Nanda	142	6	4.22% (95% CI: 0.375–0.469
Ushirika	142	10	7.04% (95% CI: 0.661–0.747
Age group (years)			
15-30	323	17	5.30% (95% CI: 0.483–0.577)
31-49	103	2	1.90% (95% CI: 0.153–0.227)

**Table 3:** Bivariate logistic regression for factors associated with the S.haematobium infection.

Variable	OR (95% CI)	*p* value
Age group (years)		
15-30	2.806 (0.637-12.353)	0.173
31-49	1	
Education		
No formal education	6.087 (2.367-15.653)	<0.001
Primary and above	1	
Occupation		
Unemployed	1.163 (0.261-5.189)	0.843
Employed	1	
Water source for domestic		
Tap water		
No	1	
Yes	0.543 (0.177-1.669)	0.287
Well		
No	1	
Yes	0.581 (0.162-2.081)	0.405
Ponds		
No	1	
Yes	0.378 (0.108-1.319)	0.127
River		
No	1	
Yes	3.374 (1.277-8.914)	0.014
Rain water		
No	1	
Yes	0.984 (0.387-2.497)	0.972
Reason for contact water		
Agriculture		
No	1	
Yes	6.020 (0.794-45.656)	0.082
Fetching water		
No	1	
	0.850 (0.189-3.823)	0.832
Fishing		
No	1	
Yes	0.700 (0.199-2.457)	0.578
Washing		
No	1	
Yes	1.127 (0.320-3.971)	0.852
Swimming		
No	1	
Yes	0.571 (0.213-1.531)	0.265
Latrine ownership		
No	1	
Yes	Unidentified	Unidentified

**Table 4:** Multivariate logistic regression models to determine the relationship of urogenital schistosomiasis and its predictors.

Variable	AOR(95% CI)	*p* value
Age group		
15-30	3.819 (0.806-18.099)	0.091
31-49	1	
Education		
No formal education	8.355 (3.035-23.001)	<0.001
Primary and above	1	
Water for domestic use		
Pond		
No	1	
Yes	3.081 (0.788-12.048)	0.106
River		
No	1	
Yes	0.386 (0.128-1.163)	0.091
Reason for contact water		
Agriculture		
No	1	
Yes	0.143 (0.18-1.126)	0.065

## Data Availability

The data supporting the conclusion are available upon request to the corresponding author.
